# Risk factors for coronary artery disease and the use of neural networks to predict the presence or absence of high blood pressure

**DOI:** 10.1186/1471-2156-4-S1-S67

**Published:** 2003-12-31

**Authors:** Catherine T Falk

**Affiliations:** 1 The New York Blood Center, 310 E. 67^th ^Street, New York, New York 10021 USA

## Abstract

**Background:**

The Framingham Heart Study was initiated in 1948 as a long-term longitudinal study to identify risk factors associated with cardiovascular disease (CVD). Over the years the scope of the study has expanded to include offspring and other family members of the original cohort, marker data useful for gene mapping and information on other diseases. As a result, it is a rich resource for many areas of research going beyond the original goals. As part of the Genetic Analysis Workshop 13, we used data from the study to evaluate the ability of neural networks to use CVD risk factors as training data for predictions of normal and high blood pressure.

**Results:**

Applying two different strategies to the coding of CVD risk data as risk factors (one longitudinal and one independent of time), we found that neural networks could not be trained to clearly separate individuals into normal and high blood pressure groups. When training was successful, validation was not, suggesting over-fitting of the model. When the number of parameters was reduced, training was not as good. An analysis of the input data showed that the neural networks were, in fact, finding consistent patterns, but that these patterns were not correlated with the presence or absence of high blood pressure.

**Conclusion:**

Neural network analysis, applied to risk factors for CVD in the Framingham data, did not lead to a clear classification of individuals into groups with normal and high blood pressure. Thus, although high blood pressure may itself be a risk factor for CVD, it does not appear to be clearly predictable using observations from a set of other CVD risk factors.

## Background

Many of the risk factors for cardiovascular disease (CVD) are now well known and widely accepted. Much of the knowledge we have today can be attributed to the very well planned longitudinal study initiated in 1948 by the National Heart Institute (now the NHLBI) in the community of Framingham, Massachusetts (the Framingham Heart Study) [1]. One goal of the study was to identify the common factors that contribute to CVD and to follow these characteristics in a longitudinal cohort over many years. The study has provided data for a variety of analyses that probably exceed the original goals (see, e.g., [2,3]).

Data from the Framingham Heart Study were provided to the participants of Genetic Analysis Workshop 13 (GAW13) and included measurements of CVD risk factors, taken over many years, on two cohorts from Framingham. The information provided did not include the diagnosis of CVD itself. In the absence of direct CVD diagnoses, we decided to look at the effect of many of the CVD risk factors on the development or presence of high blood pressure (HBP), in itself a risk factor and possibly a precursor to coronary problems. We used artificial neural networks to see if they could be trained to recognize a pattern in CVD risk factors that might lead to high blood pressure. (For obvious reasons, the blood pressure measurement itself was excluded as a risk factor.) Two separate strategies were employed. The first strategy, using data from both the original cohort and the second cohort, defined "classes" of phenotypic characteristics based on the data gathered for each individual over the course of the study, where the classes were defined based on current guidelines for "normal" or "abnormal" levels of the measurements. The second strategy used data from the second, "younger" cohort only, where fairly regular measurements were made over five time intervals for all of the phenotypic risk factors. Each time interval for each individual was considered an input record, with the outcome defined as the presence or absence of HBP in that time interval. Because of the nature of the models employed, training and validating were limited to those individuals (or records) where there was no treatment for high blood pressure. The data were "normalized" to values between 0 and 1 so that no risk factor would dominate the training of the neural network.

The first strategy resulted in a successfully "trained" network that reliably classified those in the training set as having normal or high blood pressure (BP). The validation on an independent data set was, however, not successful. The data used with Strategy 2 did not even train well (see below), suggesting that the input risk factors may not, in fact, be reliable indicators of high blood pressure.

## Methods

In the course of these analyses, two neural network programs were used for evaluation. One is NNdriver, developed in my laboratory [4]. The second is a freely available program, SNNS, distributed by the University of Stuttgart [5]. NNdriver makes use of a feed-forward back-propagation NN model and allows for multiple runs, where each run randomly divides the data into training and validation sets. Results can then be averaged over all runs to get a more accurate, representative outcome. SNNS has the option of using other NN models as well as a feed-forward back-propagation NN model. Here, we limited our runs to the same model as that employed by NNdriver in order to compare results from the two programs. We performed several runs on independently selected random samples using both programs. The results from both programs were qualitatively similar, and different randomly selected training and validation sets gave very similar results. In general the number of hidden units in the single hidden layer of the NN was set to be approximately , where *n *is the number of input values. Runs were also made with smaller or larger numbers of hidden units, but the success rate did not increase. (For a general description of the neural network models used here see the appendix of Falk et al. [4]). In the results presented below, representative runs are shown which illustrate the qualitative outcome.

### Strategy 1 (Cohorts 1 and 2)

A set of input factors was developed based on the longitudinal progression of the various risk factors. A single record was generated for each individual, based on the range and values of the risk factors. All input values were coded with a binary outcome. For example, one input factor represented the value of total cholesterol at the beginning of the study. A code of 0 (zero) was assigned if total cholesterol was <200. Otherwise a code of 1 (one) was assigned. Table 1 shows the 25 input factors used, as well as the division points between an assignment of 0 or 1.

A single output value was assigned based on whether the individual had high blood pressure at any time during the study. High blood pressure (as defined in the data set) is present if systolic BP > 140 mm Hg or diastolic BP > 90 mm Hg. Only individuals who had not been treated for high blood pressure were included. Both cohorts were coded using this strategy, resulting in a total of 574 records for Cohort 1 and 1337 records for Cohort 2. Each cohort was analyzed separately. In both cohorts the number of individuals with high and low BP were quite unbalanced. In Cohort 1 there were many more individuals with high BP than with normal BP, whereas in Cohort 2 the opposite was true. We found that simple random samples with a preponderance of high or low BP records tended to "train" well for the high frequency class and not at all well for the low frequency class. Therefore samples were selected from the total number of records available so that the number of records with normal blood pressure was approximately the same as the number of records with high blood pressure. The selected records were then randomly assigned to either the training or the validation set for each run. Both NNdriver and SNNS were used for training and validation. In the example shown below, 300 records were randomly selected for training and 300 for validating the neural network. The data are from Cohort 2.

### Strategy 2 (Cohort 2 only)

Records from all individuals in Cohort 2 were separated into five (or fewer) records, one for each time period for which measurements were made. Only time periods during which the individual was not treated for high blood pressure were included. This resulted in more than 6000 records. A single output value was assigned based on whether the individual had high blood pressure for that time period. Again, both NNdriver and SNNS were used for training and validation. In the example shown here, 300 records were randomly selected for training and 300 for validating the neural network. Runs with larger samples gave qualitatively similar results. The sample was again selected so that there was an equal number of records with normal BP and high BP. Seven input values were included in the training: sex; age; total cholesterol; fasting glucose; fasting HDL; fasting triglycerides; body mass index (BMI). All variables were "normalized" to values between zero and one so that no single factor would dominate the training.

## Results

Following training, a neural network produced a "predicted" outcome, given as a value between zero and one, for each input record. A value < 0.5 is interpreted as a prediction of normal BP and a value ≥ 0.5 as a prediction of high BP. These predicted values can then be compared to the actual classification of normal or high BP. In both of the examples shown here, 300 randomly selected individuals were used for training and 300 for validation.

### Strategy 1

**Figure 1:**
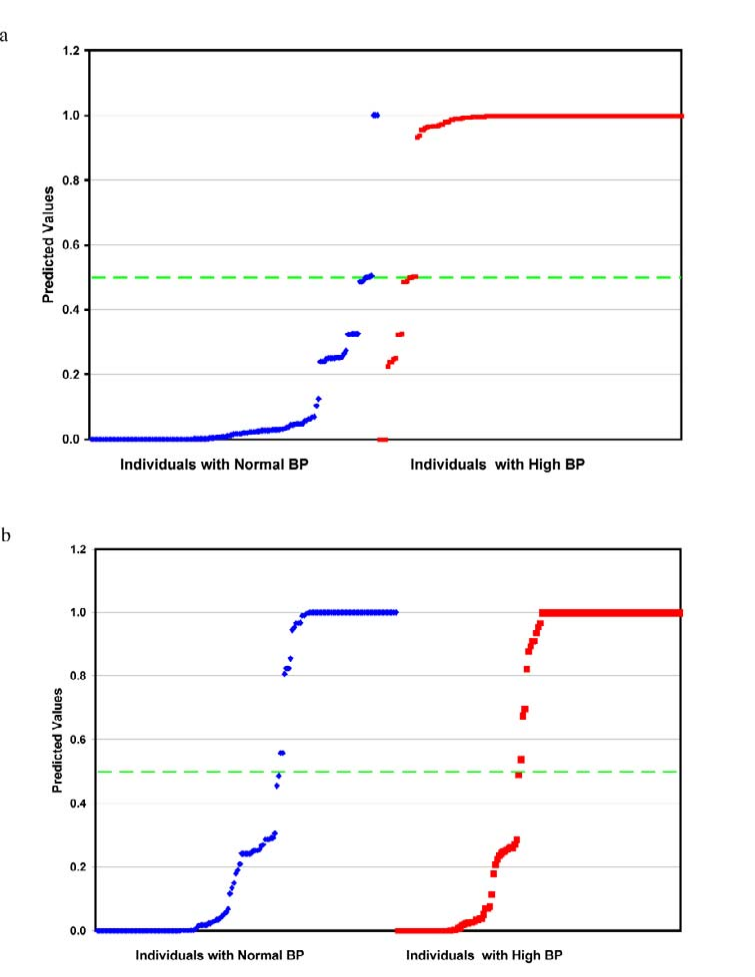
**Neural network results: Strategy 1 Cohort 2 **a, Results for the training set; b, Results for the validation set. Model with five hidden units. (Blue diamond) Normal BP.,(Red square) High BP; Y < 0.5, normal BP predicted by neural network; Y ≥ 0.5, high BP predicted by neural network.

Training using this strategy typically resulted in success rates of between 91 and 98% for the training set. Figure 1a shows the results of one run, using data from Cohort 2, where ~93% were correctly classified. The training model had five hidden units and a "learn factor" of 0.01 (in SNNS). The results show fairly good separation of the two classes. Unfortunately, the success rate of the validation set was much lower, only 59%, and the separation of the two classes was not good. This lower rate for the validation set was typical of the results for this strategy, suggesting that the trained network is not able to reliably predict the outcome for newly encountered patterns. Figure 1b shows the graph for the validation set corresponding to the training set shown in Figure 1a. As can be seen, both the high and normal BP individuals are distributed in almost "S"-shaped curves, spanning both sides of the 0.5 y-axis. This is typical of "random" predicting, where the data follow the shape of the transformation curve used in the neural network.

### Strategy 2

**Figure 2:**
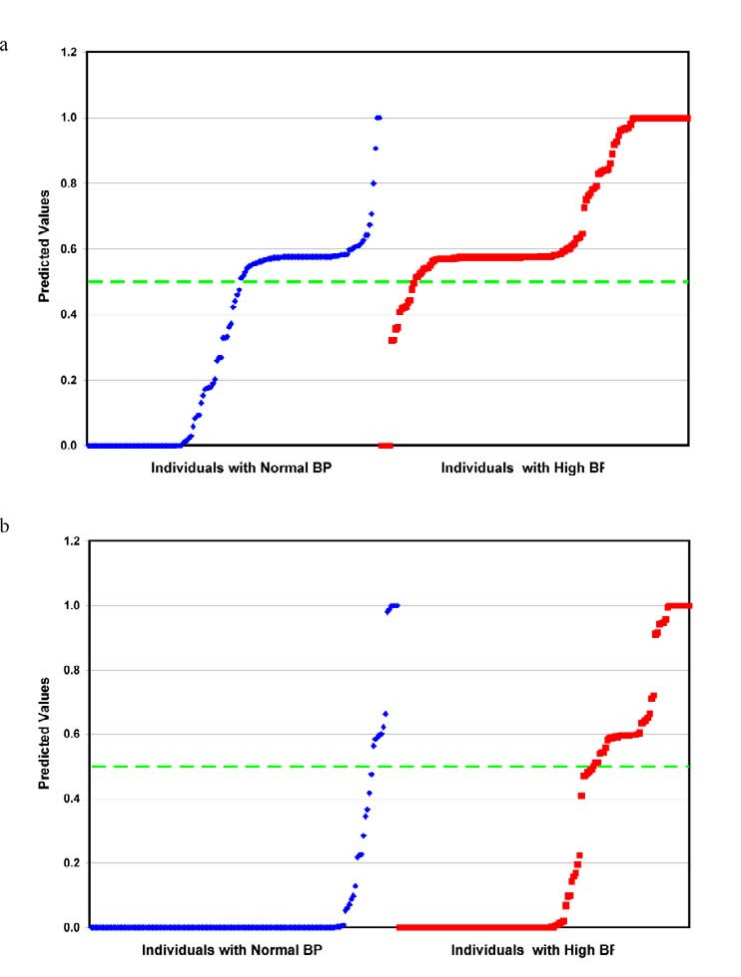
**Neural network results: Strategy 2 Cohort 2 **a, Results for the training set; b, Results for the validation set. Model with two hidden units. (Blue diamond) Normal BP.,(Red square) High BP; Y < 0.5, normal BP predicted by neural network; Y ≥ 0.5, high BP predicted by neural network.

After training the network, the training set typically predicted high or normal BP correctly between 70 and 87% of the time. However, an examination of the data shows that the data were not distinctly separated into the two classes. Figure 2a shows the distribution of results for a representative training run, where 71% of the individuals were "correctly" classified by the criterion given above. The training model had two hidden units and a "learn factor" of 0.01. It is clear that there is no real difference between the prediction curves of those with normal BP and those with high BP. In the validation set for the same run, 63% of the individuals were "correctly" classified (Figure 2b). With Strategy 2 we were unable to design a neural network that could be trained to distinctly separate those with normal BP from those with high BP.

## Discussion

Since HBP itself is considered to be a risk factor for CVD, it would be useful to have a reliable method for predicting the conditions that make one susceptible to HBP. In the absence of a defined disease status for the Framingham individuals, we chose to look at HBP as a preliminary condition to see if we could identify patterns leading to HBP. Unfortunately, the pattern of available risk factors for CVD did not provide a reliable means of predicting high blood pressure (HBP). The lack of success could be due to one of several factors, including inappropriate design of the neural networks or use of input information that does not define risk patterns leading to high BP. In the case of Strategy 1, the training results were generally quite good, but could not be validated in independent data. This suggests the possibility of over-fitting the data, i.e., using a model with too many parameters. To test the theory of over-fitting, we tested neural networks with fewer "hidden nodes", leading to a smaller parameter set. The results were essentially the same (or worse). In no case were we able to demonstrate repeatability in a validation sample. It is more difficult to determine if the input data are or are not predictive of BP status. Strategy 1 looks promising, in that the trained neural network did a good job of separating the data into high and normal BP classes. However, the failure of validation suggests that the patterns are not really describing conditions for high BP. Thus for these data, despite seemingly "good" training, the neural network did not correctly "predict" normal or high blood pressure. It is tempting to speculate that a neural network training approach would, in fact, be useful for predicting the presence of CVD, using the same set of input risk factors. Unfortunately, without direct information about CVD, we cannot say.

It is possible that another NN architecture would have been more successful in classifying records into high and normal BP classes. With SNNS one can select from a number of NN models. However, NNdriver is restricted to the feed-forward model, and we were interested in comparing results from the two programs. Limited time and this interest in comparing the two programs encouraged us to focus only on the well known feed-forward model. Another interesting extension of the study would be to compare the results of a NN analysis with those from a more conventional regression analysis. Again, time constraints made this difficult. However, another GAW13 study [6] asked a similar question about the "association" of BP status with CVD risk factors as well as with marker data. They chose to use a very different approach, namely a tree-based association model, to partition the data. Interestingly, they also failed to detect an association between high BP and the risk factors and marker data.

## Conclusions

Neural network analysis, while perhaps useful for defining subsets of individuals with correlated patterns of risk factors associated with CVD, does not seem to lead to a clear classification of individuals into groups with normal and high blood pressure. Thus, although high blood pressure may be a risk factor for CVD, it does not appear to be clearly predictable using a back propagation neural network on observations from a set of other CVD risk factors. It would be interesting to apply these methods to a sample where the CVD status were known, as the risk factors are most appropriate when used with the true disease status.

## Figures and Tables

**Table 1:** Coding of 25 Risk Factors for Strategy 1

**Category**	**Coded 0**	**Coded 1**	
Age at beginning	≥40	≥40	
Chol, beginning	≥200	≥200	
Chol, end	≥200	≥200	
Chol, change	negative	positive	
Glu minimum	<110	≥110	
Glu maximum	≥110	≥110	
LDL minimum^A^	≥130	≥130	
LDL maximum^A^	≥130	≥130	
HDL minimum	≥35	≥35	
HDL maximum	≥35	≥35	
Tri minimum	≥150	≥150	
Tri maximum	≥150	≥150	
BMI, beginning^B^	≥25	≥25	
BMI, end^B^	≥25	≥25	
BMI, change^B^	negative	positive	
Sex	female	male	

**3-column binary coding**			

cig smoked	never	always 1–20	>20 at any time
alcohol	always < 10 g	always 10–60	> 60 at any time
age at death	alive (at last measure)	≥ 50 yrs	50 yrs

^A ^LDL values were calculated from the standard formula: LDL = total cholesterol – HDL – Triglycerides/5. ^B ^BMI was calculated from the formula: BMI = 703 × Wt/(Ht)^2 ^(Units in pounds and inches).
